# Supplementary material for: On the genus Sabidius Strelzov, 1973 (Annelida: Paraonidae), with a redescription of the type species and the description of a new species
Source: PLoS One. 2020 Mar 11;15(3):e0229717. doi: 10.1371/journal.pone.0229717 (PMC7065755; doi:10.1371/journal.pone.0229717)

PLOS ONE

On the genus Sabidius Strelzov, 1973 (Annelida: Paraonidae), with a redescription of the type species and the description of a new species.

--Manuscript Draft--

**Manuscript Number:**	PONE-D-19-30964
**Article Type:**	Research Article
**Full Title:**	On the genus Sabidius Strelzov, 1973 (Annelida: Paraonidae), with a redescription of the type species and the description of a new species.
**Short Title:**	Redescription of the type species of Sabidius with a description of a new species.
**Corresponding Author:**	Natália Ranauro, MsC<br>Universidade de São Paulo<br>Sao Paulo, SP BRAZIL
**Keywords:**	"Polychaeta"; taxonomy; new species; morphology; Southwestern Atlantic Ocean
**Abstract:**	Two species of Sabidius Strelzov, 1973 were collected during a benthic survey, from 10–3,000 m deep, in Espírito Santo Basin, off southeastern Brazilian coast. Those species are S. cornatus (Hartman, 1965) and S. antennatus sp. nov. The genus Sabidius was monotypic until the present study, which describes a new species for the genus, redescribes the type species and also represents a new record for the genus in the southern Atlantic and expands the geographic and bathymetric distributions of the type species, to the southern Atlantic, at shallower depths.
**Order of Authors:**	Natália Ranauro, MsC
	Rômulo Barroso
	Paulo Cesar Paiva
	João Miguel de Matos Nogueira
**Additional Information:**	
**Question**	**Response**
**Financial Disclosure**<br><br>Enter a financial disclosure statement that describes the sources of funding for the work included in this submission. Review the submission guidelines for detailed requirements. View published research articles from *PLOS ONE* for specific examples.<br><br>This statement is required for submission and **will appear in the published article** if the submission is accepted. Please make sure it is accurate.	NR received a MsC fellowship from CAPES (Coordenação de Aperfeiçoamento de Pessoal de Nível Superior) (proc. 1586471) and currently receives a PhD fellowship from CNPq (proc. 140725/2018-0)<br>CAPES (Coordenação de Aperfeiçoamento de Pessoal de Nível Superior)<br>https://www.capes.gov.br/<br>CNPq (Conselho Nacional de Desenvolvimento Científico e Tecnológico)<br>http://www.cnpq.br/<br><br>PCP receives productivity grant from CNPq (Conselho Nacional de Desenvolvimento Científico e Tecnológico) (proc. 304321/2017-6) and FAPERJ (Fundação Carlos Chagas Filho de Amparo à Pesquisa do Estado do Rio de Janeiro) (proc. E-26/202607/2019)<br>CNPq (Conselho Nacional de Desenvolvimento Científico e Tecnológico)<br>http://www.cnpq.br/<br>FAPERJ (Fundação Carlos Chagas Filho de Amparo à Pesquisa do Estado do Rio de Janeiro) http://www.faperj.br/<br><br>JMMN receives a productivity grant from CNPq (Conselho Nacional de Desenvolvimento Científico e Tecnológico), level 2 (proc. 309599/2018-0).<br>CNPq (Conselho Nacional de Desenvolvimento Científico e Tecnológico)<br>http://www.cnpq.br/

Powered by Editorial Manager® and ProduXion Manager® from Aries Systems Corporation

**Unfunded studies**<br>Enter: *The author(s) received no specific funding for this work.*<br><br>**Funded studies**<br>Enter a statement with the following details:<br>• Initials of the authors who received each award<br>• Grant numbers awarded to each author<br>• The full name of each funder<br>• URL of each funder website<br>• Did the sponsors or funders play any role in the study design, data collection and analysis, decision to publish, or preparation of the manuscript?<br>• **NO** - Include this sentence at the end of your statement: *The funders had no role in study design, data collection and analysis, decision to publish, or preparation of the manuscript.*<br>• **YES** - Specify the role(s) played.<br><br>* typeset	We are thankful to CENPES/PETROBRAS for providing the material used for the present study. http://www.petrobras.com.br/pt/nossas-atividades/tecnologia-e-inovacao/
**Competing Interests**<br><br>Use the instructions below to enter a competing interest statement for this submission. On behalf of all authors, disclose any competing interests that could be perceived to bias this work—acknowledging all financial support and any other relevant financial or non-financial competing interests.<br><br>This statement **will appear in the published article** if the submission is accepted. Please make sure it is accurate. View published research articles from *PLOS ONE* for specific examples.	NO authors have competing interests

Powered by Editorial Manager® and ProduXion Manager® from Aries Systems Corporation

**NO authors have competing interests**<br><br>Enter: *The authors have declared that no competing interests exist.*<br><br>**Authors with competing interests**<br><br>Enter competing interest details beginning with this statement:<br><br>*I have read the journal's policy and the authors of this manuscript have the following competing interests: [insert competing interests here]*<br><br>* typeset	
**Ethics Statement**<br><br>Enter an ethics statement for this submission. This statement is required if the study involved:<br><br>• Human participants<br>• Human specimens or tissue<br>• Vertebrate animals or cephalopods<br>• Vertebrate embryos or tissues<br>• Field research<br><br>Write "N/A" if the submission does not require an ethics statement.<br><br>General guidance is provided below. Consult the submission guidelines for detailed instructions. **Make sure that all information entered here is included in the Methods section of the manuscript.**	N/A

**Format for specific study types**<br><br>**Human Subject Research (involving human participants and/or tissue)**<br>• Give the name of the institutional review board or ethics committee that approved the study<br>• Include the approval number and/or a statement indicating approval of this research<br>• Indicate the form of consent obtained (written/oral) or the reason that consent was not obtained (e.g. the data were analyzed anonymously)<br><br>**Animal Research (involving vertebrate animals, embryos or tissues)**<br>• Provide the name of the Institutional Animal Care and Use Committee (IACUC) or other relevant ethics board that reviewed the study protocol, and indicate whether they approved this research or granted a formal waiver of ethical approval<br>• Include an approval number if one was obtained<br>• If the study involved *non-human primates*, add *additional details* about animal welfare and steps taken to ameliorate suffering<br>• If anesthesia, euthanasia, or any kind of animal sacrifice is part of the study, include briefly which substances and/or methods were applied<br><br>**Field Research**<br><br>Include the following details if this study involves the collection of plant, animal, or other materials from a natural setting:<br>• Field permit number<br>• Name of the institution or relevant body that granted permission	
**Data Availability**<br><br>Authors are required to make all data underlying the findings described fully available, without restriction, and from the time of publication. PLOS allows rare exceptions to address legal and ethical concerns. See the PLOS Data Policy and FAQ for detailed information.	Yes - all data are fully available without restriction

Powered by Editorial Manager® and ProduXion Manager® from Aries Systems Corporation

A Data Availability Statement describing where the data can be found is required at submission. Your answers to this question constitute the Data Availability Statement and **will be published in the article**, if accepted.<br><br>**Important:** Stating 'data available on request from the author' is not sufficient. If your data are only available upon request, select 'No' for the first question and explain your exceptional situation in the text box.<br><br>Do the authors confirm that all data underlying the findings described in their manuscript are fully available without restriction?	
**Describe where the data may be found in full sentences. If you are copying our sample text, replace any instances of XXX with the appropriate details.**<br><br>• If the data are **held or will be held in a public repository**, include URLs, accession numbers or DOIs. If this information will only be available after acceptance, indicate this by ticking the box below. For example: *All XXX files are available from the XXX database (accession number(s) XXX, XXX.*).<br>• If the data are all contained **within the manuscript and/or Supporting Information files**, enter the following: *All relevant data are within the manuscript and its Supporting Information files.*<br>• If neither of these applies but you are able to provide **details of access elsewhere**, with or without limitations, please do so. For example:<br><br>*Data cannot be shared publicly because of [XXX]. Data are available from the XXX Institutional Data Access / Ethics Committee (contact via XXX) for researchers who meet the criteria for access to confidential data.*<br><br>*The data underlying the results presented in the study are available from (include the name of the third party*	The data are held in two polychaete collection: Museu Nacional, Universidade Federal do Rio de Janeiro and Museu de Zoologia, Universidade de São Paulo. Accesion numbers are available in the material examined in the manuscript.

and contact information or URL).<br>• This text is appropriate if the data are owned by a third party and authors do not have permission to share the data.<br><br>* typeset	
Additional data availability information:	Tick here if your circumstances are not covered by the questions above and you need the journal’s help to make your data available.

Manuscript Click here to download Manuscript Manuscript NR.docx

Full title: On the genus *Sabidius* Strelzov, 1973 (Annelida: Paraonidae), with a redescription of the type species and the description of a new species.

Short title: Redescription of the type species of *Sabidius* with a description of a new species.

NATÁLIA RANAURO[1¶*], RÔMULO BARROSO[2¶], PAULO CESAR PAIVA[3¶] & JOÃO MIGUEL DE MATOS NOGUEIRA[1¶]

[1] Laboratório de Poliquetologia (LaPol), Departamento de Zoologia, Instituto de Biociências, Universidade de São Paulo, São Paulo, SP, Brazil

[2] Instituto de Biologia, Universidade Federal da Bahia, Salvador, BA, Brazil

[3] Laboratório de Polychaeta, Departamento de Zoologia, Universidade Federal do Rio de Janeiro, Rio de Janeiro, RJ, Brazil.

[*]Corresponding author

E-mail: nataliaranauro@gmail.com (NR)

[¶] These authors contributed equally to this work.

Abstract

Two species of *Sabidius* Strelzov, 1973 were collected during a benthic survey, from 10–3,000 m deep, in Espírito Santo Basin, off southeastern Brazilian coast. Those species are *S. cornatus* (Hartman, 1965) and *S. antennatus* **sp. nov.** The genus *Sabidius* was monotypic until the present study, which describes a new species for the genus, redescribes the type species and also represents a new record for the genus in the southern Atlantic and expands the geographic and bathymetric distributions of the type species, to the southern Atlantic, at shallower depths.

Key words: ‘Polychaeta’, taxonomy, new species, morphology, Southwestern Atlantic Ocean.

Introduction

Members of the family Paraonidae Cerruti, 1909 are among the most abundant polychaetes in benthic communities, with high species richness and number of individuals [1]. Animals of this family are present in all oceans, dominant in soft bottom communities, particularly in the deep sea [2]. They are motile deposit feeders, apparently feeding on both surface and subsurface sediments [3].

Currently, there are approximately 140 species of paraonids, distributed in eight genera: *Aparaonis* Hartman, 1965 [4] (1 sp.), *Aricidea* Webster, 1879 [5] (~75 sp.), *Cirrophorus* Ehlers, 1908 [6] (7 sp.), *Levinsenia* Mesnil, 1897 [7] (22 sp.), *Paradoneis* Hartman, 1965 [4] (20 sp.), *Paraonella* Strelzov, 1973 [8] (7 sp.), *Paraonis* Cerruti, 1909 [9] (6 sp.), and *Sabidius* Strelzov, 1973 [8] (1 sp.) [10]. However, this number is

likely to be underestimated, since the family has a long history of taxonomic problems and several new species have been described recently, due to the utilization of finer meshes and the recent increase of projects focusing on the diversity of deep-sea environments [11], which are still understudied.

In spite of the number of new species described recently [12-16], for both monotypic genera, *Aparaonis* and *Sabidius*, no new species has been described since the original descriptions of *Aparaonis abyssalis* Hartman, 1965 [4] and *Sabidius cornatus* (Hartman, 1965) [4]. Although the former species is known from a single specimen (Blake 2016), members of the latter have been found in other surveys [2, 10, 17, 18].

Sabidius cornatus (Hartman, 1965) [4] was originally described as a member of *Paraonis*, but later Strelzov (1973) [8] erected the genus *Sabidius* for this species. Morphologically, these animals are similar to members of *Levinsenia*, in the absence of neuropodial lobes and medium antenna, but differ from those in having the anterior margin of prostomium with distinctively thick, tri-lobed cuticle.

Sabidius cornatus was described based on material from off New England, from slope and abyssal bottoms, between 400–2,900 m deep [4, 17]. Posteriorly, members of this species were found among material from the western Pacific [2], along the U.S Atlantic slope, from the Canadian boundary to off the Carolinas [18], and from slope depths in the Gulf of Mexico, off Louisiana [10].

Regarding the Brazilian coast, few taxonomic studies on the Paraonidae have been conducted [19-21]. Previous studies reported the presence of five genera and 30 species of Paraonidae from Brazilian shoreline, most of them from shallower waters [22].

The aim of this study is to describe a new species of *Sabidius* and re-describe *S. cornatus*, including amendments on the geographic and bathymetric ranges of the latter,

based on type material and specimens collected from off southeastern Brazil, state of Espírito Santo, 19°3'S 37°44'W–21°10'S 38°28'W. The present is the first record for the genus *Sabidius* in the Southern Atlantic Ocean.

Material and methods

The specimens were collected off the state of Espírito Santo, 19°3'S 37°44'W–21°10'S 38°28'W, southwestern Atlantic, during a survey conducted and coordinated by CENPES/PETROBRAS (Brazilian Energy Company), under the scope of the AMBES project (“Caracterização Ambiental Marinha da Bacia do Espírito Santo e Porção Norte da Bacia de Campos”).

The oceanographic surveys were completed on board of the oceanographic vessels Seward Johnson and Luke Thomas, from December 2010 to July 2013. Sampling was performed at four different areas: mouth of Rio Doce; continental shelf; slope and canyons (Fig 1). A total of 20 sampling sites were chosen in the area in front of the mouth of Rio Doce, from 10–51 m deep. Sampling was performed along seven transects from south to north. Each transect was composed of ten sampling stations, four of which in the continental shelf, at 25, 40, 50, and 150 m deep, respectively, and six along the slope, at 400, 1000, 1300, 1900, 2300 and 3000 m deep, comprising 70 sampling sites. Sampling campaigns were also made at Cânion Watu Norte (CANWN) and Cânion Rio Doce (CAND), along depth gradient, 150, 400, 1000, and 1300 m deep. Samples were collected triplicately, by means of a 294 l van Veen grab or a 125 l Box-Corer. Each of the four areas was sampled twice, once in summer and again in winter.

Fig 1. This is the Fig 1 Title. This is the Fig 1 legend. Study area; sampling stations of Project AMBES at four different areas: mouth of Rio Doce; continental shelf; slope and canyons.

Identifications were based on specific morphological characters. Specimens were examined under stereomicroscope, compound light microscope and scanning electron microscope (SEM). For the SEM, specimens were dehydrated in a series of progressively increasing concentrations of ethanol (70–100%), then critical point dried (LEICA EM CPD300), coated with ~35 nm of gold and examined and photographed at Laboratório de Microscopia Eletrônica (NEMA/PUC-Rio), Laboratório de Miscroscopia Eletrônica do Museu Nacional (UFRJ) and Laboratório de Microscopia Eletrônica, Instituto de Biociências, Universidade de São Paulo (IB–USP).

Description of the new species was based on all the specimens examined, data of the holotype is provided inside parentheses, after the range of variation observed for each character. Only complete specimens were measured in length; width was measured at chaetiger 4.

Specimens are deposited in the Museu Nacional, Universidade Federal do Rio de Janeiro (MNRJ) and Museu de Zoologia, Universidade de São Paulo (MZUSP).

Nomenclatural Acts

The electronic edition of this article conforms to the requirements of the amended International Code of Zoological Nomenclature, and hence the new names contained herein are available under that Code from the electronic edition of this article. This published work and the nomenclatural acts it contains have been registered in ZooBank,

the online registration system for the ICZN. The ZooBank LSIDs (Life Science Identifiers) can be resolved and the associated information viewed through any standard web browser by appending the LSID to the prefix “http://zoobank.org/”. The LSID for this publication is: urn:lsid:zoobank.org:pub: XXXXXXX. The electronic edition of this work was published in a journal with an ISSN, and has been archived and is available from the following digital repositories: PubMed Central, LOCKSS.

Results

Systematics

Family Paraonidae Cerruti, 1909

Genus *Sabidius* Strelzov, 1973

Type species. *Paraonis cornatus* Hartman, 1965.

Diagnosis (emended after Blake, 2016 [10]). Body long and slender with numerous segments, easily broken. Prostomium with distinctive thick, tri-lobed cuticle on anterior margin; median antenna present or absent; ciliated bands, terminal sensory organ and cheek organs all absent; curved nuchal organs on posterior margin of prostomium, often fused to each other, extending across dorsum. Three prebranchial segments present; branchiae restricted to few segments. Neuropodia without podial lobes; notopodial lobes may be present on posterior body notopodia. Chaetae include slender notopodial capillaries, curved modifed spines on postbranchial neuropodia, of

which at least 1–2 per fascicle with long arista. Pygidium with three digitiform anal cirri.

Remarks. The generic diagnosis is emended above, to allow for the presence of a median antenna, as observed among members of *S. antennatus* **n. sp.** (see below), which is absent in *S. cornatus*.

***Sabidius cornatus* (Hartman, 1965)**

(Figs 2–4)

Paraonis cornatus Hartman 1965: 140–141; Hartman & Fauchald 1971: 102.

Sabidius cornatus. Strelzov 1979: 179–180.

Material examined. Type series. North Atlantic, USA, New England, Continental slope, upper end of canyon just west of Atlantis Canyon, anchor dredge, R/V ATLANTIS Sta. Slope 4, coll. Sanders, H., Woods Hole Oceanographic Institution, 2. Holotype (LACM-AHF POLY 655) incomplete, 5.4 mm long, 0.2 mm wide, coll. 28 August 1962, 39°56’30’’N 70°39’54’’W, 400 m. Paratype: 52 specs (LACM-AHF POLY 656), coll. 28 August 1962, 39°56’30’’N 70°39’54’’W, 400 m.

Material examined. 2 specs. (MZUSP xxxx), coll. 21º04'09.61"S 40º13'7.38"W, 396 m, 30 Dec 2011; 3 specs. (MZUSP xxxxxx), coll. 9 Jan 2012, 20º14'19.45"S 39º48'36.67"W, 416 m; 1 spec. (MZUSPxxxxx), coll. 11 Dec 2011, 19º33'20.99"S 39º02'36.2"W, 374 m; 1 spec. (MZUSPxxxxx), coll. 14 Jan 2012, 19º49'37.21"S 39º35'41.25"W, 352 m deep; 2 specs. (MZUSPxxxx), coll. 14 Jan 2012, 19º46'34.99"S 39º30'04.65"W, 402 m; 3 specs. (MZUSPxxxx), coll. 14 Dec 2011, 19º36'26.24"S

39º10'17.35"W, 352 m deep; 2 specs. (MZUSPxxxx), coll. 9 Dec 2011, 19º34'20.42"S 38º41'18.43"W, 438 m; 2 specs. (MZUSPxxxx), coll. 19 Jun 2013, 20º14'17.95"S 39º48'34.35"W, 438 m; 2 specs. (MZUSPxxxx), coll. 30 Jun 2013, 19º34'20.47"S 38º41'19.8"W, 438 m.

Diagnosis. Prostomium without median antenna, nuchal organs curved [illegible]ts on posterior margin associated with two areas of reddish-brown pigmentation often fused and extending across dorsum. Three prebranchial segments; branchiae short, narrow. Notopodia and neuropodia both lacking podial lobes. Notochaetae as slender capillaries; neurochaetae including enlarged, curved, modified spines, of which at least 1–2 per fascicle with long arista, on postbranchial segments. Pygidium as expanded lobe, anal cirri not observed [10].

Description. All specimens incomplete, with 24–66 chaetigers. Body long and slender, fragile, easily broken. Preserved specimens white–yellowish, without pigmentation patterns other than prostomial spots. Prostomium longer than wide, with Prostomium with distinctive thick, tri-lobed cuticle on anterior margin (Fig 2A–[illegible] median antennae, ciliated bands, terminal sensory organ, and cheek organs all absent; nuchal organs curved slits on posterior margin (Fig 2A, C) associated with reddish–brown pigments often fused to each other and extending across dorsum (Fig 3A). Eyes absent. Ventral mouth with saclike pharynx, everted in some specimens. Three prebranchial chaetigers (Fig 2A–[illegible]Six to seven pairs of distinctly short, cirriform, dorsal branchiae; first four pairs less than 40 µm long, last three pairs slightly longer (Fig 2B), more than 40 µm long; inner margin of branchiae basally ciliated, continuing through ciliary band across dorsum, connecting filaments within pairs [illegible]g 2D). First three chaetigers wider than long, following chaetigers as wide as long (Fig 2A–B). Parapodia biramous and papillated, [illegible]oto- and neuropodial lobes both absent (Fig 2B),

notochaetae emerging dorso-laterally, neurochaeta emerging at lateral edges. Notochaetae all capillary, arranged in single row; prebranchial chaetigers with 5 notochaetae chaetae each; branchial and postbranchial chaetigers with 4 chaetae per fascicl[illegible]ig 2A–B). Neurochaeta of three types: capillary chaetae, curved modified spines (Fig 2E) and curved modified spines with long arista (Fig 3B); neurochaetae always arranged in single rows; prebranchial chaetigers with 5 capillary neurochaetae each; branchial chaetigers with 7 capillary neurochaetae each; postbranchial chaetigers, until chaetiger 11, with 4 capillary chaetae, from chaetiger 12 to end of body, neurochaetae as 5–6 enlarged, curved modified spines per chaetiger (Fig 2E), of which at least 1–2 with long arista. Pygidium not observed, all specimens incomplete.

Fig 2. This is the Fig 2 Title. This is the Fig 2 legend. [illegible]*bidius cornatus* (A–B). Anterior chaetigers, dorsal and lateral views, respectively; (C). Prostomium, frontal-dorsal view; (D). Pair of branchiae, anterior view; (E). Curved modified spines. br = branchiae; sb = scar of branchiae; no = nuchal organ.

Fig 3. This is the Fig 3 Title. This is the Fig 3 legend. [illegible]*idius cornatus* (Holotype LACM-AHF POLY 655) (A). Anterior chaetigers, dorsal view; (B). Neuropodium with 2 curved modified spines and 1 with long arista.

Habitat. Found in muddy sediments, with small amount of sand, 10.3–15.7°C.

Distribution. Northern Atlantic Ocean: U.S Atlantic slope from off the Canadian boundary to the Carolinas [18], type locality off New England, from slope to abyssal depths, 400–2,946 m deep [4, 17]; slope depths off Louisiana, Gulf of Mexico [10]. Southern Atlantic Ocean: Southeastern Brazil, off Espírito Santo, 352–438 m (Fig 4). Pacific Ocean: east off Hokkaido Island, 3,388 m deep [2].

Fig 4. This is the Fig 4 Title. This is the Fig 4 legend. Samplings of the species of *Sabidius* in summer (above) and winter (bellow). Numbers in legend refers to abundance at each station.

Remarks. The specimens herein examined match both the original description of this species (Hartman, 1965), based on New England material, and the re-description, based on material from the Atlantic and Pacific Oceans [2]. Although both the original description and the re-description by Strelzov [2] did not mention the slight difference in branchial length along branchiate chaetigers, this character was confirmed when the type material was examined. Eyes were not visible neither in Brazilian specimens nor in type material, although they were described as present in former descriptions [2, 4]). However, those are not true eyes but pigment spots instead [2], which were described as eyes by Hartman [4], while Strelzov [2] stated the eyes are not always conspicuous because the pigmentation readily fades after preservation. Branchial cilia were not mentioned either by Hartman [4] or Strelzov [2], however this character is only visible under the SEM and that technique was not available by the time of those descriptions.

Specimens of *S. cornatus* were not found in habitats deeper than 438 m, although the survey which collected the material used for the present study sampled bottoms down to 3,000 m deep. This is the shallowest and the most restricted bathymetrically report for this species, within a range of 100 m only. Northern Atlantic material was obtained from slope and abyssal depths, within a bathymetric range of 2,500 m, and apparently those animals are more abundant in deeper bottoms [17].

***Sabidius antennatus* n. sp.**

(Figures 4–6)

Material examined. Type series. Holotype (MNRJP–1497) complete, 4.25 mm long, 0.12 mm wide, coll. 19 January 2012, 19º43'14.34"S 39º33'34.86"W, 45 m. Paratypes: 3 specs (MNRJP–1492), coll. 15 Dec 2010, 19º49'57.38"S 39º52'14.02"W 29 m; 7 specs (MNRJP–1496), coll. 15 Jan 2012, 19º45'54.56"S 39º30'25.23"W, 121 m; 4 specs (MNRJP–1494), coll. 22 Jan 2012, 21º3'27.14"S 40º22'59.61"W, 36 m; 4 specs (MNRJP–1495), coll. 21 Jan 2012, 20º34'53.42"S 40º06'27.43"W 45 m; 1 spec. (MNRJP–1493), coll. 9 Dec 2011, 19º31'51.66"S 39º03'04.04"W, 140 m. All type specimens from off Espírito Santo, southeastern Brazil.

Additional material. State of Espírito Santo: 2 specs (MZUSP xxxx), coll. 22 jan 2012, 21º04'01.29"S 40º18'50.11"W, 46 m; 2 specs (MNRJP–1499), coll. 21 Jan 2012, 20º34'32.47"S 40º20'52.37"W, 20 m; 1 spec. (MNRJP–1500), coll. 21 Jan 2012, 20º35'25.16"S 39º54'58.31"W, 145 m; 1 spec. (MNRJP–1501), coll. 20 Jan 2012, 20º11'25.35"S 40º02' 16.02"W, 35 m; 2 specs (MNRJP–1502), coll. 19 Jan 2012, 19º43'14.34"S 39º33'34.86"W, 45 m; 1 spec. (MZUSP xxxx), coll. 19 Jan 2012, 19º26'05"S 39º17'38.92"W, 46 m; 2 specs (MNRJP–1498), coll. 15 Jan 2012, 19º36'04.32"S 39º10'34.07"W, 134 m; 1 spec. (MNRJP–1503), coll. 18 Jan 2012, 18º52'32.61"S 39º08'42.82"W, 34 m; 2 specs (MNRJP–1504), coll. 18 Jan 2012, 18º53'29.72"S 39º06'23.3"W, 43 m; 1 spec. (MNRJP–1485), coll. 17 Dec 2010, 19º52'21.52"S 39º59'33.54"W, 26 m; 1 spec. (MNRJP–1486), coll. 12 Dec 2010, 19º35'12.39"S 39º38'33.16"W, 29 m; 1 specimen (MNRJP–1487), coll. 16 Dec 2010, 19º57'32.89"S 39º53'30.69"W, 43 m; 1 spec. (MNRJP–1488), coll. 12 Dec 2010, 19º37'48.27"S 39º35'25.83"W, 38 m; 2 specs (MNRJP–1489), coll. 16 Dec 2010, 20º01'03.73"S 39º50'13.76"W, 48 m; 1 spec. (MNRJP–1490), coll. 16 Dec 2010,

19º55’44.66”S 39º45’38.7”W, 46 m; 5 specs (MNRJP–1491), coll. 12 Dec 2010, 19º41’33.92”S 39º31’17.74”W, 48 m; 3 specs (MNRJP–1505), coll. 17 Jan 2012, 18º40’55.3”S 38º55’41.48”W, 44 m; 19 specs (MZUSP xxxx), coll. 11 Jul 2013, 21º03’31.13”S 40º22’59.88”W, 34 m; 1 spec. (MZUSP xxxx), coll. 11 Jul 2013, 21º04’04.56”S 40º14’14.08”W, 147 m; 2 specs (MZUSP xxxx), coll. 12 Jul 2013, 20º34’34.37”S 40º20’50.77”W, 21 m; 1 spec. (MZUSP xxxx), coll. 12 Jul 2013, 20º34’47.13”S 40º11’31.1”W, 34 m; 8 specs (MZUSP xxxx), coll. 12 Jul 2013, 20º34’53.05”S 40º06’27.68”W, 43 m; 1 spec. (MZUSP xxxx), coll. 13 Jul 2013, 20º35’23.09”S 39º55’01.18”W, 156 m; 6 specs (MZUSP xxxx), coll. 13 Jul 2013, 20º11’25.75”S 40º02’15.87”W, 33 m; 24 specs (MZUSP xxxx), coll. 13 Jul 2013, 20º12’21.46”S 39º58’00.3”W, 45 m; 1 spec. (MZUSP xxxx), coll. 14 Jul 2013, 19º43’14.33”S 39º33’34.78”W, 43 m; 4 specimens (MZUSP xxxx); coll. 27 Jun 2013, 19º45’53.43”S 39º30’25.97”W, 138 m; 2 specs (MZUSP xxxx), coll. 15 Jul 2013, 19º18’06.12”S 39º23’23.35”W, 33 m; 1 spec. (MZUSP xxxx), coll. 29 Jun 2013, 19º36’03.57”S 39º10’33.64”W, 43 m; 1 spec. (MZUSP xxxx), coll. 15 Jul 2013, 18º52’31.35”S 39º08’41.34”W, 33 m; 2 specs (MZUSP xxxx), coll. 16 Jul 2013, 18º53’31.97”S 39º06’21.78”W, 43 m; 3 specs (MZUSP xxxx), coll. 17 Jul 2011, 19º57’32.36”S 39º53’33.01”W, 46 m; 4 specs (MZUSP xxxx), coll. 16 Jul 2011, 19º52’29.66”S 39º49’08.1”W, 41 m; 1 spec. (MZUSP xxxx), coll. 15 Jul 2011, 19º42’26.81”S 39º39’05.27”W, 36 m; 3 specs (MZUSP xxxx), coll.15 Jul 2011, 19º37’41.83”S 39º35’31.52”W, 35 m; 4 specs (MZUSP xxxx), coll. 17 Jul 2011, 20º01’02.6”S 39º50’18.72”W, 49 m; 2 specs (MZUSP xxxx), coll. 16 Jul 2011, 19º55’45.59”S 39º45’41.35”W, 43 m; 4 specs (MZUSP xxxx), coll. 15 Jul 2011, 19º50’16.39”S 39º40’11.23”W, 46 m; 3 specs (MZUSP xxxx), coll. 13 Jul 2011, 19º41’24.99”S 39º31’20.42”W, 44 m; 1 spec. (MZUSP xxxx), coll. 13 Jul 2011,

19º32’28.16”S 39º41’35.15”W, 11 m; 4 specimens (MZUSP xxxx), coll. 16 Jul 2011, 19º49’52.15”S 39º52’24.51”W, 28 m; 1 spec. (MZUSP xxxx), coll. 15 Jul 2011, 19º44’44.06”S 39º46’32.01”W, 29 m; 2 specs (MZUSP xxxx), coll. 22 Jan 2012, 21º03’27.14”S 40º22’59.61”W, 36 m; 2 specs (MZUSP xxxx), coll. 21 Jan 2012, 20º34’53.42”S 40º06’27.43”W, 45 m; 2 specs (MZUSP xxxx), coll. 16 Dec 2010, 19º57’32.89”S 39º53’30.69”W, 43 m; 2 specs (MZUSP xxxx), coll. 12 Dec 2010, 19º37’48.27”S 39º35’25.83”W, 38 m; 1 spec. (MZUSP xxxx), coll. 15 Jan 2012, 19º45’54.56”S 39º30’25.23”W, 121 m; 1 spec. (MZUSP xxxx), coll. 15 Jan 2012, 19º36’04.32”S 39º10’34.07”W, 134 m; 4 specs (MZUSP xxxx), coll. 21 Jan 2012, 20º34’32.47”S 40º20’52.37”W, 20 m; 1 specimen (MZUSP xxxx), coll. 18 Jan 2012, 18º52’32.61”S 39º08’42.82”W, 34 m.

Diagnosis. Prostomium with median antenn[illegible]hort mid–dorsal tubercule on first chaetiger. Three prebranchial chaetigers; six pairs of well–developed dorso-lateral branchiae, each chaetiger one pair per chaetiger. Notopodial lobes only developed on posterior chaetigers; neuropodial lobes absent throughout. Pygidium with 3 short digitiform anal cirri.

Description. Complete specimens with 50–77 chaetigers (77), 4.47–5.83 mm (5.22) in length, 0.09–0.16 mm (0.13 mm) wide. Body long and slender, fragile, easily broken. Preserved specimens white–yellowish, without pigmentation patterns other than prostomial spots. Prostomium longer than wide, with distinctive thick, tri-lobed cuticle on anterior margin; median antenna present, 0.03–0.05 mm (0.05 mm) in length, not reaching anterior margin of prostomium, digitiform with round expanded tip (Figs 5A–D; 6A); ciliated bands, terminal sensory organ, and cheek organs all absent; nuchal organs curved slits on posterior margin of prostomium associated with light brown pigments often fused to each other and extending across dorsum (Figs 5A–B; 6A). Eyes

absent. Ventral mouth with saclike pharynx, everted in some specimens (Fig 5D). Three prebranchial chaetigers. Six pairs of well-developed, digitiform and distally tapered branchiae, filaments progressively longer, last pairs twice as long as first pairs, or longer (Figs 5C, E–G; 6A); filaments of 1st pair 40–60 µm (45 µm) long, those of 2nd pair 50–70 µm (55 µm), 3rd pair 60–80 µm (70 µm), 4th pair 70–90 µm (80 µm), 5th pair 80–120 µm (105 µm), and 6th pair 90–140 mm (115 mm) long; branchial filaments heavily ciliated at bases, connected to each other across dorsum within pairs by ciliary bands (Fig 5C–G). Chaetiger 1 wider than long, with short mid–dorsal tubercule (Figs 5A–D; 6A) following chaetigers almost as wide as long, last 10 chaetigers wider than long. Parapodia biramous and papillated, notochaeta emerging dorso-laterally, neurochaeta at lateral edges, notopodial lobes present on last ~10 chaetigers (Fig 5J); neuropodial lobes absent throughout. Notochaeta all capillary, arranged in single row; prebranchial chaetigers with 8 notochaetae each, branchial segments with 4 notochaetae per fascicle, postbranchial chaetigers with 3. Neurochaeta always in single row, of 3 types: capillary chaetae, curved modified spines (Figs 5H–J; 6B, C) and curved modified spines with long arista (Figs 5H; 6B); neurochaetae always arranged in single rows; prebranchial chaetigers with 10 capillary neurochaetae each; branchial chaetigers with 7 capillary neurochaetae each; postbranchial chaetigers, until chaetiger 12, with 6 capillary chaetae, from chaetiger 13 to end, neurochaetae as 5–7 enlarged curved modified spines (Figs 5H, I; 6B, C), at least 1–2 of which per fascicle with long arista (Fig 5H, J; 6B). Pygidium with three digitiform anal cirri along ventral margin, all about same length (Fig 5K; 6C).

Fig 5. This is the Fig 5 Title. This is Fig 5 legend. *Subidius antennatus* sp. nov. (A–B). Prostomium, dorsal view; (C). Anterior chaetigers, dorsal view; (D). anterior chaetigers

dorso-lateral view; (E). Branchial chaetigers, dorsal view; (F). Pair of branchiae, antero-lateral view; (G). Close up of one branchia, lateral view; (H). Neuropodium with 7 curved modified spines and 1 with long arista; (I). Curved modified spines; (J). Posterior chaetigers, ventral view; (K). Pygidium, ventral view. at = antenna; ac = anal cirri; br = branchiae; fbr = first branchiae; lbr = last branchiae; nl = notopodial lobe; no = nuchal organ; ph = everted pharynx; st = short tubercule.

Fig 6. This is the Fig 6 Title. This is Fig 6 legend. [illegible]*idius antennatus* **n. sp.** (A). Anterior chaetigers, dorsal view; (B). Neuropodium with 5 curved modified spines and 1 with long arista; (C). Last chaetigers, ventral view.

Habitat. Found in muddy-sand sediments, with few scattered boulders, 13–25.6°C.

Distribution. Southern Atlantic Ocean: southeastern Brazil, off Espírito Santo state, 11–156 m deep (Fig 4).

Remarks. Individuals of *S. antennatus* **n. sp.** are very similar morphologically to specimens of the other known species of this genus, *S. cornatus*. Animals of both species share the presence of a distinctive thick, tri-lobed cuticle on the anterior margin of the prostomium, similar types of modified chaetae, few pairs of branchiae, and the absence of neuropodial lobes. These two species, however, are differentiated because members of *S. antennatus* **n. sp.** have a median antenna and longer and more heavily ciliated branchial filaments.

We consider the morphology of prostomium, with crested anterior margin, the numbers of prebranchial and branchial chaetigers, and the morphology of branchia with a pair of filaments at either side of chaetigers, the diagnostic characters for members of *Sabidius*. Therefore, the presence of a medial antenna is variable within the

genus, as also occurs among members of three other genera of paraonids, *Aricidea* [23], *Cirrophorus* and *Paradoneis* [15].

Members of *S. antennatus* **n. sp.** also differ from individuals of *S. cornatus* in having a short mid–dorsal tubercule on 1st chaetiger and notopodial lobes on posteriormost chaetigers, but it is doubtful whether posteriormost chaetigers of members of *S. cornatus* were ever examined, as the specimens are very fragile and a complete specimen has never been registered.

The presence of a short mid-dorsal tubercule on 1st chaetiger is a unique characteristic among Paraonidae. No other species of the family has this tubercule on 1st chaetiger. The closest feature present within the family is a large mid-dorsal rounded papilla on first branchial segment of *Aricidea* (*Strelzovia*) *claudiae* Laubier, 1967 [24].

The pygidium of members of *S. cornatus* was not described neither in the original description of the species [4], nor in the re-description by Strelzov [2], due to the absence of complete specimens in both studies (as also herein). Blake [10] was the first to describe complete specimens and, according to the author, the pygidium has an extended smooth lobe, while among members of all other genera of paraonids there are 2–3 anal cirri; due to the fragility of these animals, it is possible that Blake [10] described a damaged pygidium. Among members of *S. antennatus* **n. sp.**, the pygidium has 3 digitiform anal cirri along the ventral margin, all of similar length.

Specimens described as *Aricidea* (*Acmira*) sp. C from northeastern Brazil, off Maranhão State, in a PhD thesis [21] match the description of *Sabidius antennatus* **n. sp.** Specimens from northeastern Brazil were obtained from fine sand bottoms, 28–28.30°C, between 33–55 m depth. Although we did not examine those specimens from off Maranhão, [illegible] strongly believe the specimens belong to *S. antennatus* **n. sp.**, due to the presence of antenna, same distribution of branchiae and size and shape of modified

chaetae**.** Before expanding the distribution of *S. antennatus* **n. sp.** for the northeastern Brazil, however, those specimens should be examined.

As regards to the bathymetric distribution, individuals of *S. antennatus* inhabit shallower bottoms, at continental shelf depths.

Etymology. The specific name refers to the presence of a medial antenna, which is herein described for the first time for members of *Sabidius*.

Acknowledgments

We are thankful to CENPES/PETROBRAS for providing the material used for the present study.

References

1. Hilbig B, Blake J. Deep-sea polychaete communities in the northeast Pacific Ocean off the Gulf of the Farallones, California. Bulletin of Marine Science. 2016; 78 (2): 243–269.
2. Strelzov VE. Polychaete worms of the family Paraonidae Cerruti, 1909 (Polychaeta, Sedentaria). English translation of the 1973 Russian edition, published by the Smithsonian Institution and U.S. National Science Foundation, Washington D.C.; 1979.
3. Jumars PA, Dorgan KM, Lindsay SM. Diet of Worms Emended: An Update of Polychaete Feeding Guilds. Annual Review of Marine Science. 2015; 7: 497–520. doi: 10.1146/annurev-marine-010814-020007.

4. Hartman O. Deep-water benthic polychaetous annelids off New England to Bermuda and other North Atlantic areas. Occasional Papers of the Allan Hancock Foundation. 1965; 28: 1–384.

5. Webster HE. The Annelida Chaetopoda of the Virginian coast. Transactions of the Albany Institute. 1879; 9, 202–269.

6. Ehlers E. Die Bodensässigen Anneliden aus den Sammlungen der deutschen Tiefsee-Expedition. In: Chun C, editor. Wissenschaftliche Ergebnisse der deutschen Tiefsee-Expedition auf dem Dampfer 'Valdivia' 1898–1899, Vol.16. Gustav Fischer, Jena; 1908. pp 1–168. doi: 10.5962/bhl.title.2171

7. Mesnil F. Études de morphologie externe chez les Annélides. II. Remarques complémentaires sur les Spionidiens. La famille nouvelle des Disomidiens. La place des Aonides (sensu Tauber, Levinsen). Bulletin scientifique de la France et de la Belgique. 1897; 30: 83–100.

8. Strelzov VE. Polychaete worms of the family Paraonidae Cerruti, 1909 (Polychaeta, Sedentaria). Akademiya Nauk SSSR, Leningrad; 1973.

9. Cerruti A. Contributo all'anatomia, biologia e sistematica delle Paraonidae (Levinsenidae) con partiolare riguardo alle specie del golf di Napoli. Mittheiluungen aus der Zoologischen Station zu Neapel. 1909; 19 (3): 459–512.

10. Blake JA. Paraonidae Cerruti, 1909. *In*: Westheide W, Purschke G. editors. *Handbook of Zoology, Annelida: Polychaetes*. Gruyter, Berlin; 2016. pp. 6–52.

11. Aguirrezabalaga F, Gil J. Paraonidae (Polychaeta) from the Capbreton Canyon (Bay of Biscay, NE Atlantic) with the description of eight new species. Scientia Marina. 2009; 73 (4): 631–666. doi: 10.3989/scimar.2009.73n4631.

12. Arriaga-Hernández S, Hernández-Alcántara P, Solís-Weiss V. Description and distribution of two new species of Paraonidae (Annelida: Polychaeta) from a lagoon-

estuarine ecosystem in the Southern Gulf of Mexico. Zootaxa. 2013; 3686 (1): 051–064. doi: 10.11646/zootaxa.3686.1.2.

13. Çinar ME, Dağli E. Polychaetes (Annelida: Polychaeta) from the Aegean and Levantine coasts of Turkey, with descriptions of two new species. Journal of Natural History. 2013; 47 (13–14): 911–947. doi: 10.1080/00222933.2012.752543.

14. Erdoğan-Dereli D, Çinar ME, Dağli E. *Cirrophorus* species (Annelida:Polychaeta: Paraonidae) in the Sea of Marmara, with description of a new species. Zootaxa. 2017; 4347 (1): 137–150. doi: 10.11646/zootaxa.4347.1.8.

15. Langeneck J, Busoni G, Aliani S, Castelli A. Deep-sea polychaetes (Annelida) from the Malta Escarpment (western Ionian Sea). The European Zoological Journal. 2017; 84 (1): 142–152. doi: 10.1080/24750263.2017.1287964.

16. Magalhães WF, Bailey-Brock JH, Barroso R. A new species of *Paradoneis* Hartman, 1965 (Annelida: Paraonidae) from Hawaii with notes on its reproductive strategy. Marine Biodiversity. 2019; 49 (4):1617–1625. doi: 10.1007/s12526-018-0929-y.

17. Hartman O, Fauchald K. Deep-water benthic polychaetous annelids off New England to Bermuda and other North Atlantic areas. Part II. Allan Hancock Monographs in Marine Biology. 1971 6: 1–327.

18. Blake JA, Hecker B, Grassle JF, Brown B, Wade M, Boehm P., et al. Study of biological processes on the U.S. South Atlantic slope and rise. Phase 2. U.S. Department of the Interior, Minerals Management Service, Washington, D.C. 1987; 1–403. Available from: http://marinecadastre.gov/espis/#/search/study/9007.

19. Bolivar GA**.** Orbiniidae, Paraonidae, Heterospionidae, Cirratulidae, Capitellidae, Maldanidae, Scalibregmidae e Flabelligeridae (Annelida: Polychaeta) da costa

sudeste do Brasil (22°57’S - 27°20’S). PhD Thesis, Universidade Federal do Paraná, Brasil. 1990.

20. Barroso R. Anelídeos poliquetas das famílias Amphinomidae, Opheliidae, Syllidae e Paraonidae de oceano profundo da Bacia de Campos, Sudeste do Brasil. PhD Thesis, Museu Nacional Universidade Federal do Rio de Janeiro, Brasil. 2011.

21. Franklin-Junior W. Anelídeos poliquetas da plataforma continental da costa nordeste semiárida: estados do Ceará, Piauí e Maranhão, na região entre o Rio Coreaú e o Delta do Parnaíba. PhD Thesis, Universidade Federal do Ceará, Brasil. 2015.

22. Amaral ACZ, Nallin SAH, Steiner TM. Catálogo das espécies de Annelida Polychaeta do Brasil. Available from: http://www.ib.unicamp.br/museu_zoologia/files/lab_museu_zoologia/Catálogo_Polychaeta_Brasil_Amaral_et_al_2013_1a.pdf.

23. López E, Sikorski A. The Paraonidae (Annelida: Sedentaria) from Norway and adjacent seas, with two new species, four new records, and a redescription of Paraonides nordica Strelzov, 1968 based on type material. Zootaxa. 2017; 4320 (1): 041-067. doi:10.11646/zootaxa.4320.1.3.

24. Laubier L. Sur quelques Aricidea (Polychètes, Paraonidae) de Banyuls-sur-Mer. Vie et Milieu 1967; 18(1-A): 99-132.

Figure 1 Click here to download Figure Fig 1.tif

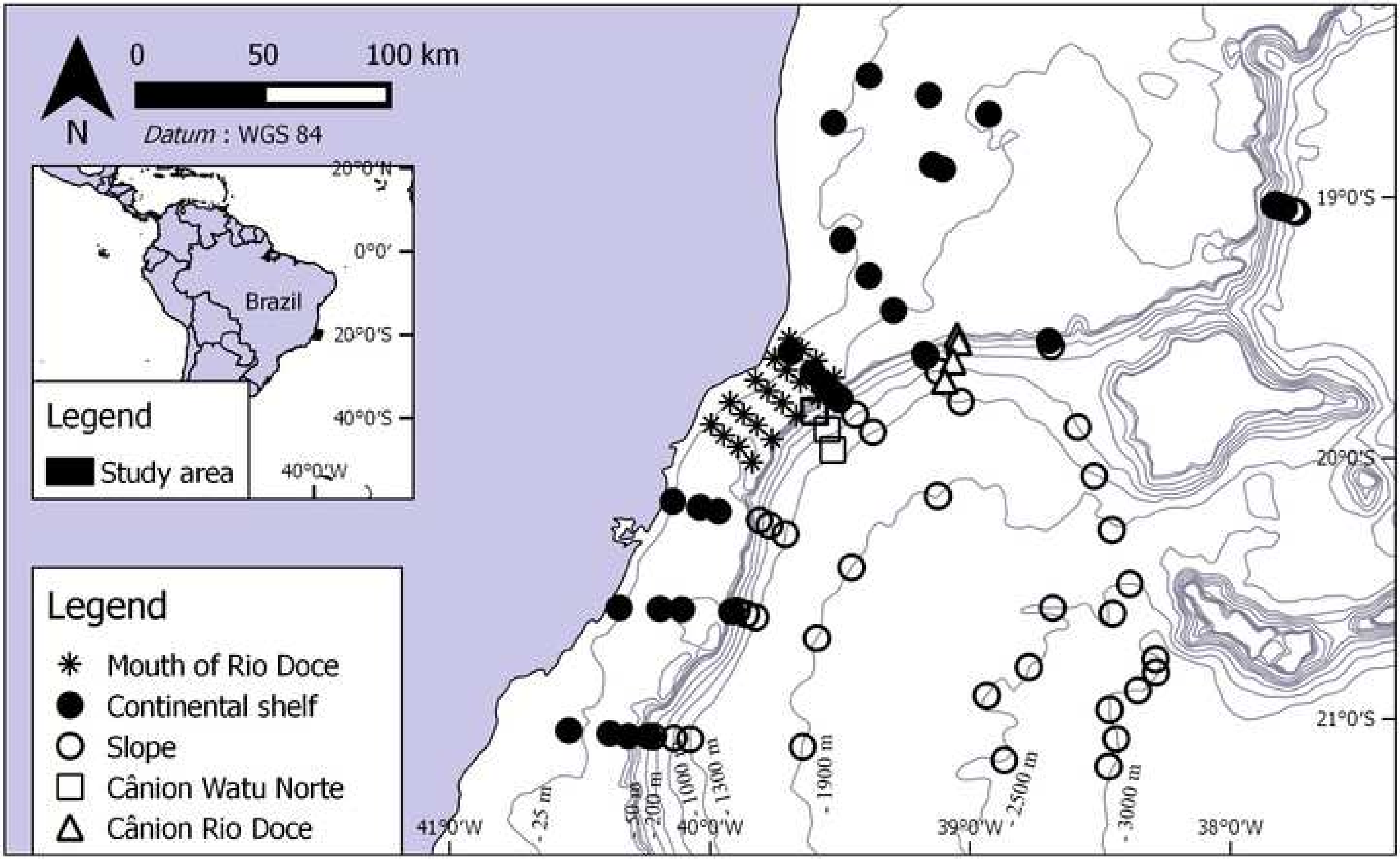

Figure 2

Click here to download Figure Fig 2.tif

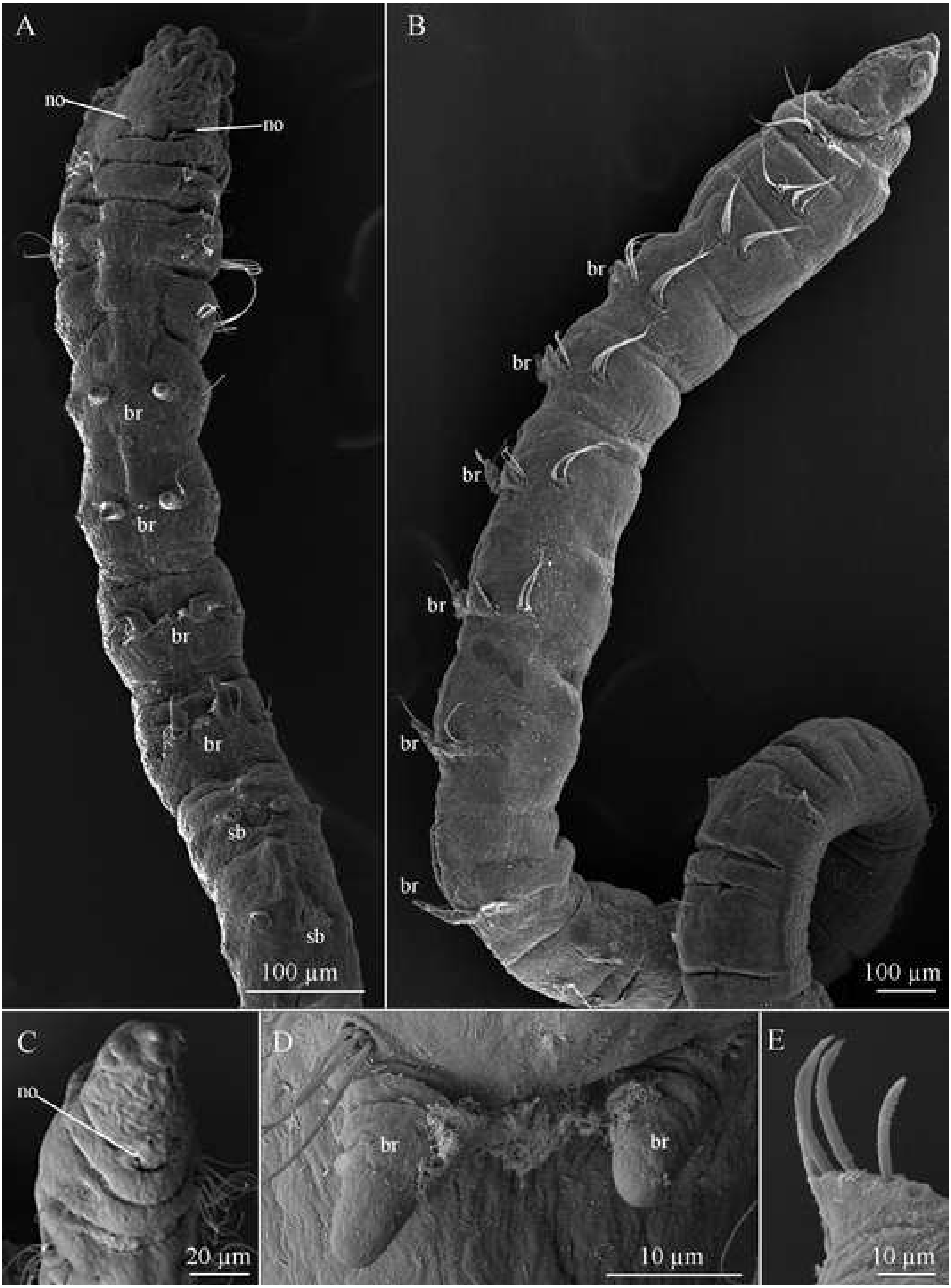

Figure 3

Click here to download Figure Fig 3.tif

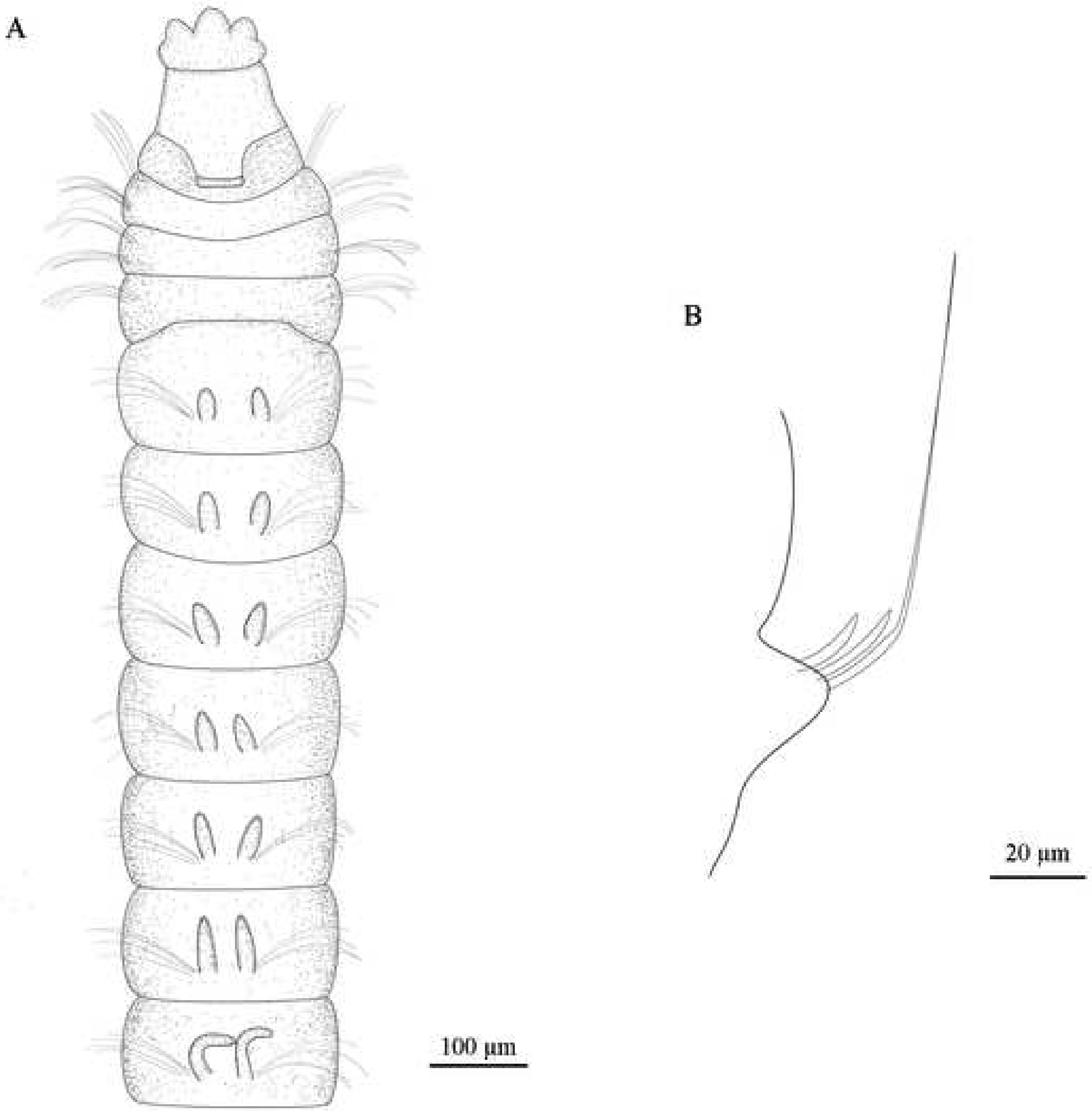

Figure 4

Click here to download Figure Fig 4.tif

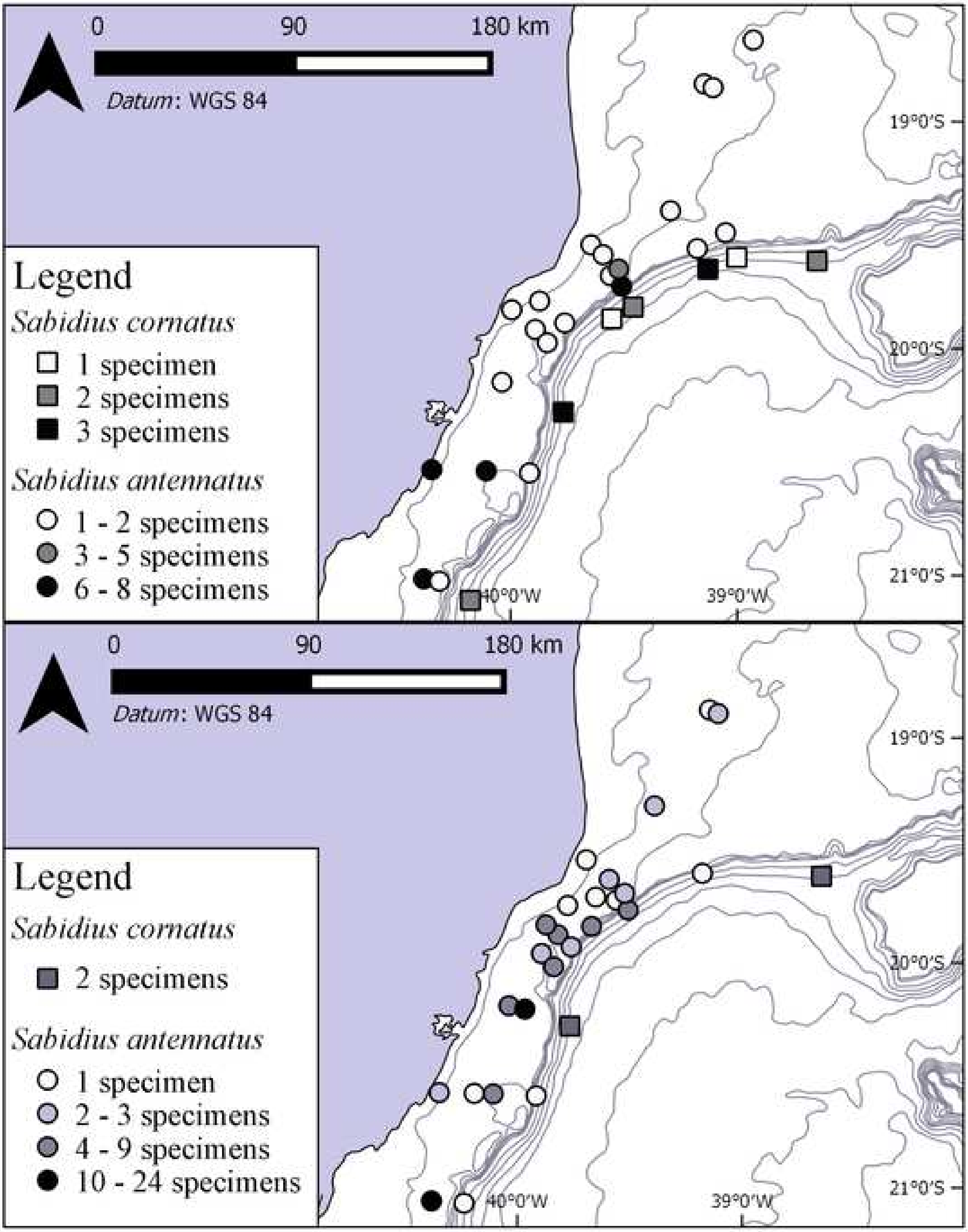

Figure 5

Click here to download Figure Fig 5.tif

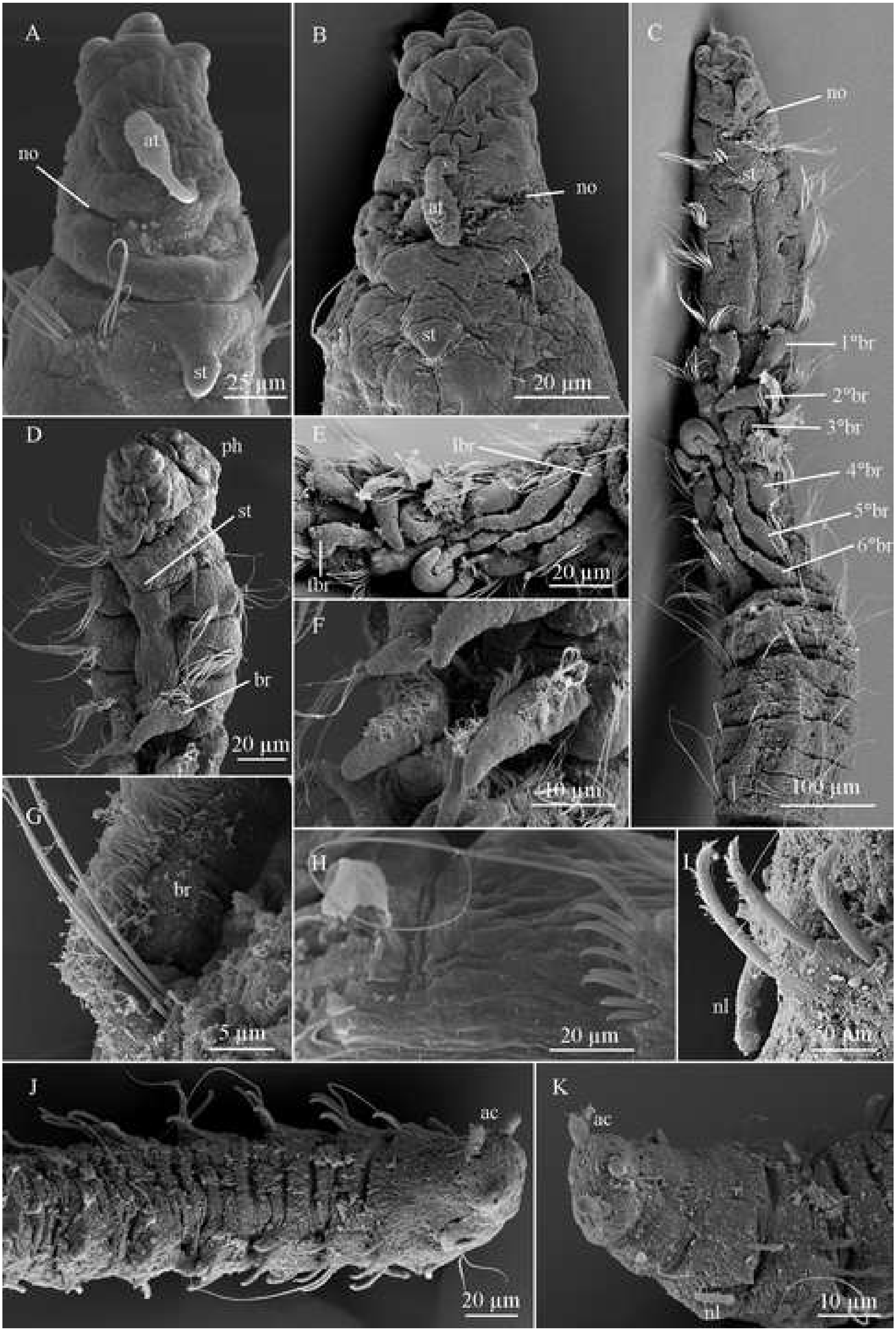

Click here to download Figure Fig 6.tif

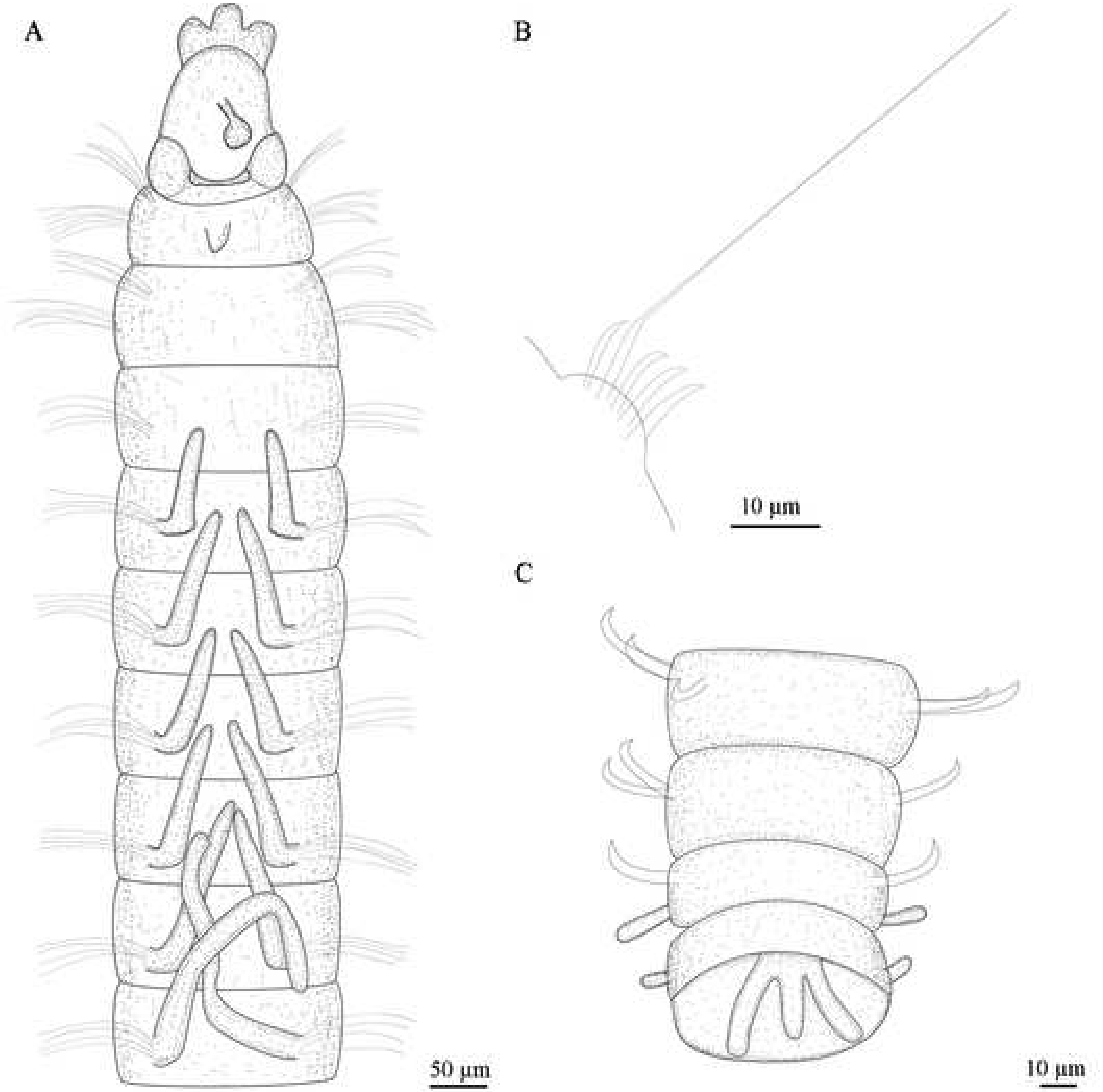

Supplement: Attachment — Submitted filename: PONE-D-19-30964_reviewer-comments.pdf [file pone.0229717.s001.pdf]
