# Supplementary material for: On the genus Sabidius Strelzov, 1973 (Annelida: Paraonidae), with a redescription of the type species and the description of a new species
Source: PLoS One. 2020 Mar 11;15(3):e0229717. doi: 10.1371/journal.pone.0229717 (PMC7065755; doi:10.1371/journal.pone.0229717)
Supplement: Attachment — Submitted filename: Response to Reviewers.docx [file pone.0229717.s003.docx]

Dear Editor & Reviewers,

Thank you very much for the review of the manuscript. All the suggestions were taken in consideration. Please find bellow a list showing how we dealt with each point raised by the academic editor and reviewers of the manuscript by Natália Ranauro; Rômulo Barroso; Paulo César Paiva; and João Miguel de Matos Nogueira, titled " On the genus Sabidius Strelzov, 1973 (Annelida: Paraonidae), with a redescription of the type species and the description of a new species".

We hope the manuscript is now acceptable for publication in PLOS One

Thank you very much for your kindness. Yours sincerely,

Natália Ranauro

**Review Comments to the Author**

Reviewer 1: The goals of the paper are plenty achieved with the material studied, the methods applies and the results obtained; however, their importance is only of limited reach. Without considering whether it merits publication in this journal or in any other directly devoted to taxonomy, some flaws must be fixed before acceptance. First of all, English language needs a thorough revision in the abstract and discussion headings. Also figures need some reworking; whereas map are of good quality and SEM photographs are superb, ink drawings are of poor quality and need to be worked out.

**NR:** One of the main goals in zoology is to understand the relationships between different metazoan taxa. Researchers estimate that only half of the total diversity of annelids has been described. Only describing new species we can fully understand the phylogenetic relationships within each phylum. Describing new species and new morphological features can help to create new hypothesis of phylogenetic relationships within the family Paraonidae and in the Annelida phylogeny. For the family Paraonidae, most of the specimens available in the museums were fixed with formalin, what makes hard gene extraction for molecular analysis. This highlights the importance of morphological research and description of new species. English language has been reviewed. The drawings were modified, although they loose quality when converted into pdf format.

**Reviewer 1**

Modifications are marked in green.

**C1:** A through revision of the English language is needed (not only for the abstract). Within the abstract give diagnostic features of the genus and the two species.

**NR:** A careful revision of the English language was made. The diagnostic features of the genus and the two species were included in the abstract.

**C2:** Not appreciable in figure

**NR:** I agree that the papillated nature of parapodia are not appreciable in figure 2B, but at least between chaetigers 2-6 it is possible to see the the papillated nature of neuropodia. The figure 2A is showing not only the papillated parapodia, but also the biramous feature of the parapodia and the absent of both noto- and neuropodial lobes.

**C3:** Apparently more in ink drawing

**NR:** Ink drawing was modified.

**C4:** Quality of figure 3A can be (and should be) improved

**NR:** Quality of figure 3A was improved.

**C5:** The quality of these drawings must be also enhanced

**NR:** The drawings were modified and enhanced.

**C6:** Define the character in a more clear way

**NR:** The morphology of the branchiae has been described.

**Review Comments to the Author**

Reviewer 2: The manuscript provides a redescription of the type species of the polychaete genus *Sabidius* and description of a new species from Brazil. In general, it is well-written, the morphological characters used to designate the new species are well-defined and appropriate considering the current knowledge on paraonids, and the species are illustrated with both line drawings and SEM images. However, I have provided suggestions to improve the reading and the descriptions. One major edition that the authors should follow is the style of the description. It is common practice in taxonomy that the descriptions are based on the holotypes and the variation should be reported for the paratypes (either within parentheses or within the text).

**NR:** The style of the description was modified as suggested.

**Reviewer 2**

Modifications are marked in yellow

**C1:** Too wordy. Suggestion:

The genus *Sabidius* was monotypic until the present study, in which a new species is proposed together with a redescription of the type species with expansion of the geographic distribution of the genus and of the type species.

**NR:** Suggestion accepted.

**C2:** Could it belong to a complex of species?

**NR:** Due to the wide bathymetric and geographic distribution of this species we do not exclude the possibility this species is part of a complex of species, although more studies are needed to confirm this hypothesis, including molecular data, which we could not access for this paper, as all the specimens examined were preserved in formalin solution.

**C3:** Descriptions should be based on holotype specimen. The variation within parentheses should be of paratypes.

**NR:** Descriptions were modified as suggested by the reviewer.

**C4:** Is this based on the literature? Why at chaetiger 4?

**NR:** The literature usually uses the largest chaetiger to measure the specimens, but ‘the largest chaetiger’ could be variable, due to preservation artifacts. We decided to measure width at chaetiger 4 for all specimens, because it is where branchiae begin.

**C5:** Indicate using text in italics which sections of the diagnosis were emended.

**NR:** Suggestion accepted.

**C6:** Comment here that the presence/absence of a median antenna is most likely a homoplastic character in paraonids, given that it has been used to either separate genera and species within a genus. e.g. your species of *Sabidius*, genera *Paradoneis* and *Cirrophorus*.

**NR:** Comment included.

**C7:** How many chaetigers?

**NR**: 35 chaetigers. Information added on manuscript.

**C8:** Are all paratypes incomplete?

**NR:** Yes. Information was added on manuscript.

**C9:** Without arista??

**NR:** Neurochaetae of three types: capillary chaetae, curved modified spines without arista and curved modified spines with long arista.

**C10:** Pygidium was described in the diagnosis. Absent or present (but lacking cirri)??

**NR:** The pygidium was described in the diagnosis provided by Blake 2016. The type material and the specimens from Brazil were incomplete. Blake (2016) described the pygidium as an expanded lobe without pygidial cirri but since such type of pygidium has never been described for any Paraonidae species, we believe Blake described an incomplete or damaged specimen. This problem is raised in the discussion.

**C11:** Include depth here.

**NR:** Depth included.

**C12:** Type locality should be presented first then the observed geographic distribution of the species.

**NR:** Type locality presented first.

**C13:** Add the number of segments of the holotype, even if it is an anterior fragment.

**NR:** The number of chaetigers was added.

**C14:** The description should be of the holotype and the range presented should be of the paratypes.

**NR:** The description was reorganized as suggested.

**C15:** Either use simple curved modified spines or curved modified spines without arista.

**NR:** We used curved modified spines without arista.

**C16:** How many specimens of each species were used for SEM? Could this ciliation on the dorsum be variable among individuals of the same species?

**NR:** Three specimens of each species were examined under the SEM. The pattern of ciliation was consistent among different specimens. We do not believe this pattern of ciliation on dorsum could be variable among individuals of the same species.

**C17:** This paragraph should be included in the Remarks of the genus

**NR:** The paragraph was included in the Remarks of the genus.

**C18:** But see your diagnosis

**NR:** The diagnosis presented in the paper is the based on the diagnosis of Blake (2016) with the proper emendation. None of the type specimens described by Hartman (1965) is complete and the pygidium was not described. Blake (2016) made this statement for the pygidium based on non-type material. We believe that Blake described the ‘pygidium’ based on an incomplete or damaged specimen, and that’s the reason for him having described a pygidial morphology which is not consistent with any other known species of Paraonidae (and was not observed by us in complete specimens of *S. antennatus*).
